# Oxidative stress mediates the association between triglyceride-glucose index and risk of cardiovascular and all-cause mortality in metabolic syndrome: evidence from a prospective cohort study

**DOI:** 10.3389/fendo.2024.1452896

**Published:** 2024-08-20

**Authors:** Ming Yang, Qing Shangguan, Guobo Xie, Guotai Sheng, Jingqi Yang

**Affiliations:** Department of Cardiovascular Medicine, Jiangxi Provincial People’s Hospital, The First Affiliated Hospital of Nanchang Medical College, Nanchang, Jiangxi, China

**Keywords:** triglyceride-glucose index, cardiovascular, all-cause mortality, metabolic syndrome, oxidative stress, mediation effect

## Abstract

**Background:**

The aim of this study was to investigate the relationship between triglyceride-glucose (TyG) index and cardiovascular disease (CVD) and all-cause mortality in adults with metabolic syndrome (MeS) and explore the mediating role of oxidative stress.

**Methods:**

This study included 6131 adults with MeS from the National Health and Nutrition Examination Survey (NHANES). The relationships between TyG index and mortality were elucidated using multivariate Cox proportional hazards models, restricted cubic splines (RCS) Fine-Gray competing risk model. In addition, mediation analysis was used to test the indirect effect of oxidative stress indicators.

**Results:**

Over a median 106-month follow-up, a total of 357 CVD and 1292 all-cause deaths were recorded. After multivariate adjustment, there was a J-type relationship between TyG index and CVD and all-cause mortality, with optimal inflection point of 9.13 and 8.92. After the threshold point, TyG index was positively associated with CVD (HR: 4.21, 95%CI: 1.82, 9.78) and all-cause mortality(HR: 2.93, 95%CI: 2.05, 4.18). Even using non-cardiovascular mortality as a competitive risk, the Fine-Gray model also illustrated that the cumulative CVD mortality incidence was higher in MeS with TyG index >9.13 (Fine-Gray P< 0.01). Mediation analysis revealed that biomarkers of oxidative stress, including gamma-glutamyl transferase and uric acid, collectively mediated 10.53% of the association between the TyG index and CVD mortality, and 8.44% of the association with all-cause mortality (P < 0.05).

**Conclusion:**

In the cohort study, TyG index was found to have a J-shaped association with CVD mortality and all-cause mortality in MeS population and oxidative stress may play a key mediating role in this relationship.

## 1 Introduction

Metabolic Syndrome (MetS) represents a confluence of metabolic abnormalities that has become a global health concern, affecting an estimated 30% of the adult population worldwide (1). MeS, which included elevated blood glucose, hypertension, dyslipidemia, and abdominal obesity, was a significant risk factor for cardiovascular diseases (CVD) (2). The prevalence of CVD in MetS patients is alarming, with studies suggesting that individuals with MetS are at least twice as likely to develop CVD compared to those without the syndrome (3). Moreover, the risk of all-cause mortality is heightened in this population, with a reported increase of 1.5 times (3, 4), suggesting the urgency for effective therapeutic strategies to reduce these risks.

The triglyceride-glucose (TyG) index, a novel biomarker measured by fasting blood glucose (FBG) and triglycerides (TG), has emerged as a potent predictor of insulin resistance (5) and lipid metabolism disorders (6). Several studies have shown that TyG index significantly correlates with the risk of CVD and major cardiovascular events and has considered to be a valuable tool for risk stratification and prognosis assessment (7–11). Despite this, studies on the metabolic syndrome population and the corresponding mechanisms remain difficult to elucidate.

Oxidative stress, a key mediator of cellular damage and inflammation, has been implicated in the pathophysiology of MetS (12). It has been found that in people with MetS, oxidative stress may play an important role through pathways involving endothelial dysfunction, pro-inflammatory cytokine production, and mitochondrial damage (13–15). Varying degrees of insulin resistance may be present in metabolic syndrome populations, and TyG, a marker of insulin resistance, may influence the prognosis of these populations through oxidative stress.

Therefore, this study aims to elucidate the relationship between TyG and the risk of CVD and all-cause mortality in individuals with MetS, with a particular focus on the mediating role of oxidative stress. By unraveling these complex interactions, we hope to contribute to the clinical management of MetS and lay the foundation for future research aimed at improving patient prognosis.

## 2 Methods

### 2.1 Study design and participants

The prospective cohort study utilized data from the National Health and Nutrition Examination Survey (NHANES, https://www.cdc.gov/nchs/nhanes/index.html), a nationally representative survey conducted by the Centers for Disease Control and Prevention (CDC). All surveys were reviewed and approved by the Ethics Review Committee of the National Centre for Health Statistics and all participants provided written informed consent. The data used in this study were all from the 1999-2018 NHANES database. The entire research process strictly followed the STROBE reporting guidelines (**Supplementary Table 1**).

**Figure 1:**
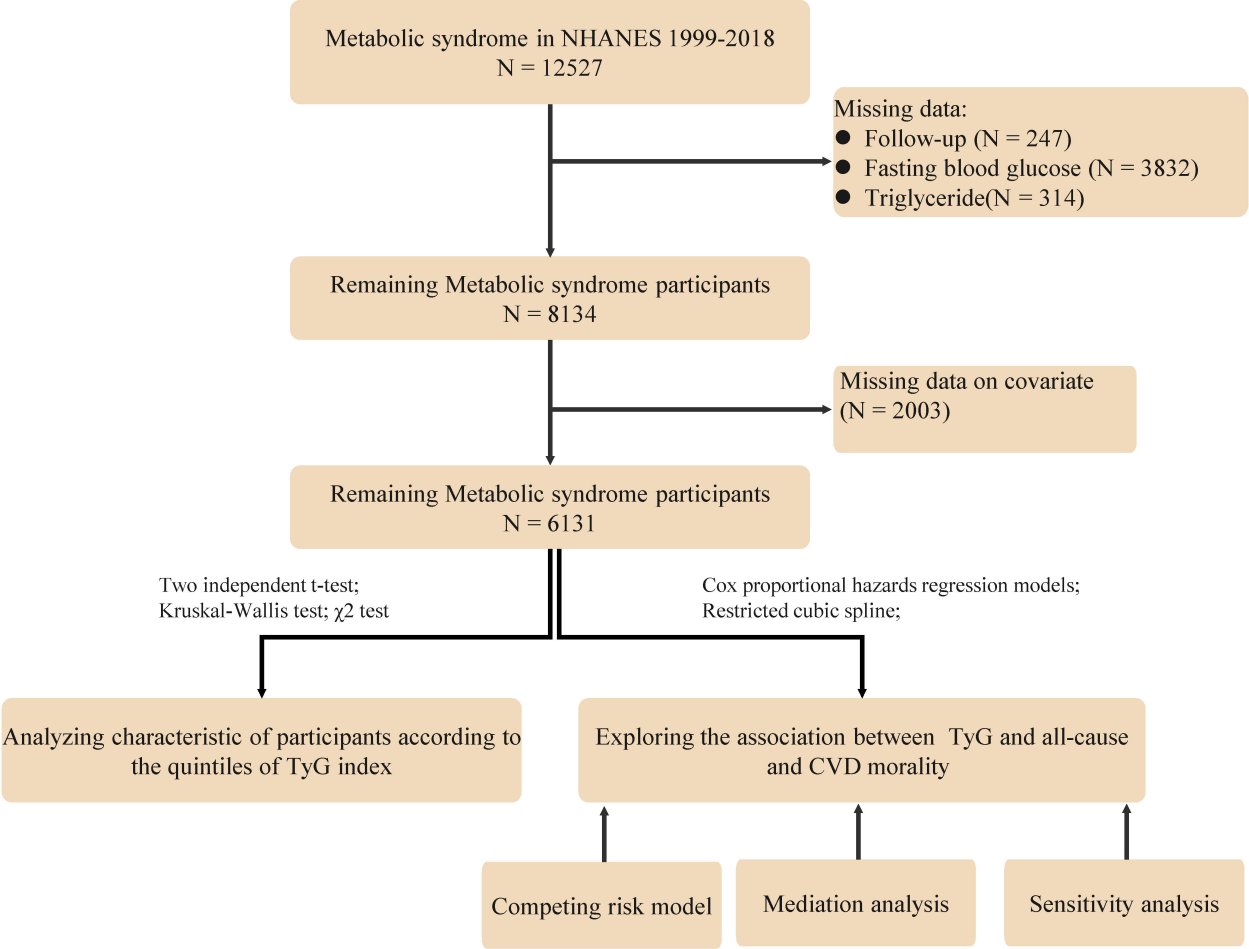
Study flowchart.

The study population consisted of adults who were diagnosed with MetS based on the National Cholesterol Education Program Adult Treatment Panel III (NCEP ATP III) criteria (16), which is the presence of at least three of the following conditions: (1) Waist circumference ≥102 cm for male and ≥88 cm for female; (2) Fasting serum triglycerides ≥150 mg/dL; (3) High-density lipoprotein (HDL) cholesterol <40 mg/dL for male and <50 mg/dL for female; (4) Blood pressure ≥130/85 mmHg or current use of medication for hypertension; (5) Fasting blood glucose ≥ 100 mg/dl or current use of medication for hyperglycemia. Specific exclusion criteria were as follows (1): Participants under the age of 18; (2): Pregnant participants; (3) Participants lacking data on blood glucose, lipids and follow-up; and (4) Missing data on other covariates. For the population with missing data, since the data were missing at random, we followed the approach of previous studies by excluding them from subsequent analyses. After screening, 6131 people with MeS were finally included (**Figure 1**).

### 2.2 Study variables

The TyG index was calculated using the formula (17): Ln[fasting triglyceride (mg/dL) × fasting glucose (mg/dL)/2]. Fasting blood samples (at least 8 hours or more but less than 24 hours) were collected and analyzed for triglycerides and glucose levels using standard laboratory techniques.

Oxidative stress as a mediating variable, including gamma-glutamyl transferase (GGT) and uric acid. The quantification of GGT activity was performed using a modified version of the assay initially described by Szasz in 1969 (18). The assay involves the enzymatic conversion of L-gamma-glutamyl-3-carboxy-4-nitroanilide to 5-amino-2-nitrobenzoate and L-gamma-glutamyl-glycylglycine in the presence of glycylglycine, catalyzed by GGT. Uric acid is oxidized by uricase and the peroxide produced from this reaction is acted upon by peroxidase in the presence of 4 aminophenazone to produce a measurable colored product. A detailed description of the laboratory methods used can be found in the Laboratory Methods Documentation section of NHANES(https://www.cdc.gov/nchs/nhanes/index.html).

All-cause mortality was based on vital status and cause of death information for participants tracked by the National Center for Health Statistics before 31 December 2019. Mortality outcomes were ascertained by linking the NHANES data with the National Death Index (NDI). Cardiovascular (I00-I09, I11, I13, I20-I51) were defined according to the International Classification of Diseases, Tenth Clinical Revision (ICD-10) system codes and the NCHS classified heart diseases (054-064). Detailed definitions and categorization can be found at: https://www.cdc.gov/nchs/data/datalinkage/underlying-and-multiple-cause-of-death-codes-508.pdf. We extracted potential leading causes of death from the mortality profile and the all-cause mortality and CVD mortality were used as endpoint events.

### 2.3 Assessment of covariates

In our investigation, we accounted for a range of potential confounding variables that might impact the study outcomes. A comprehensive set of sociodemographic and lifestyle characteristics were identified through structured interviews and physical assessments conducted by trained personnel. These variables included age(years), gender (male or female), racial/ethnic classification (Mexican-American, Other Hispanic, non-Hispanic white, non-Hispanic black, and other), educational attainment categorized as less than high school, high school diploma or equivalent, and college education or higher, as well as the family poverty income ratio, which was stratified into ≤1, >1 to ≤3, and >3. Lifestyle habits, including smoking status (never smoked, current smoker, and former smoker) and alcohol intake frequency (never, daily or nearly daily, 3 to 4 times per week, 1 to 2 times per week, and less than once per week), were also documented. Anthropometric measurements, such as body mass index (BMI), were obtained from physical examinations conducted at a mobile examination facility. Laboratory measurements included aspartate serum creatinine (Scr), total cholesterol (TC), and low-density lipoprotein cholesterol (LDL). The estimated glomerular filtration rate (eGFR) was estimated using the updated chronic kidney disease epidemiology collaboration equation (CKD-EPI) (19) and was reported in mL/min/1.73 m^2^. Additionally, personal medical histories, including hypertension, diabetes, heart failure, stroke, coronary heart disease, angina, and myocardial infarction, were recorded. Information on medication use was also collected, encompassing antihypertensive medications, diuretics, and statins.

### 2.4 Statistical analysis

R software (version 4.3.0) and Empower software (version 4.1) were used to analyze the study data. Participant characteristics at baseline were presented using medians and interquartile ranges for continuous data and percentages for categorical data. Continuous variables were assessed using the t-test or Kruskal-Wallis test, while categorical variables were assessed using the chi-squared test. TyG index quartiles were defined with the first quartile (Q1) being the lowest and the fourth quartile (Q4) being the highest. Cox proportional hazards regression models were used to explore the relationship between TyG index and mortality. The Schoenfeld residual method is a type of residual used to test whether the residual terms of a Cox model are time-dependent. If the P-value of the test is >0.05 suggesting that the residual is stochastically related to time, it means that the proportional risk assumption is met. Then, three models were constructed, each with different levels of covariate adjustment: Model 1 (unadjusted), Model 2 (adjusted for age and race), and Model 3 (further adjusted for education, poverty income ratio, alcohol consumption, smoking status, total cholesterol, LDL, eGFR, heart failure, stroke, coronary heart disease, angina, myocardial infarction, oral antihypertensive medications, diuretics, and statins). To examine the association between TyG index and mortality, we used 4-knot(0.05, 0.35, 0.65, 0.95) restricted cubic spline(RCS) and smooth curve fitting (penalized spline method). In nonlinear relationships, we used segmented Cox regression models on both side of the effect point to investigate the relationship between TyG index and the mortality. Furthermore, in order to comprehensively assess the relationship, the “tidycmprsk” package was used to perform the Fine-Gray competing risk model. The Fine-Gray test was utilized to analyze non-CVD mortality as a competing risk and to compare the relationship between the TyG index and CVD mortality before and after the inflection point.

To investigate the potential mediating role of oxidative stress in the relationship between TyG index and mortality outcomes, we performed a mediation analysis using the bootstrap method with 5000 iterations. Based on previous reports, we chose GGT and uric acid as markers of oxidative stress. The mediation model was structured to estimate the indirect effects of oxidative stress markers on the relationship between TyG index and mortality. The proportion of the effect of TyG index on mortality mediated by oxidative stress markers was quantified as the ratio of the indirect effect to the total effect. Initially, we evaluated the mediating roles of GGT and uric acid separately in the relationship between the TyG index and mortality. Subsequently, we assessed the combined mediation effect of GGT and uric acid. In addition, Bootstrap testing was employed to ascertain the significance of the mediation effect (20). This method involves creating numerous bootstrap samples by resampling the original dataset with replacement. For each bootstrap sample, the mediation analysis was performed to calculate the indirect effect-the influence of the independent variable on the dependent variable via the mediator. The significance of the mediation was evaluated by examining the proportion of bootstrap estimates that crossed a predetermined threshold, commonly zero, which signifies no effect.

We conducted a sensitivity analysis to test the robustness of our findings. We performed Cox proportional hazard regression separately on the population with MeS complicated by CVD, on the population excluding those who died within two years and on the population who were taking oral antihypertensive medications, diuretics, or statins to eliminate their confounding effects. To further test the robustness of our analysis of the association between TyG and mortality in MeS patients, we calculated an E-value (21) based on the effect size of TyG based on the Model 3 to quantify the minimum strength of association a confounder would need with the study outcome. Two-sided P-value <0.05 were considered statistically significant.

## 3 Results

### 3.1 Baseline characteristics of study participants

A total of 6131 subjects with MeS were finally included in our study(**Figure 1**), of which 47.74% were male, with an average age of 57.42 ± 15.60 years. During a median follow-up of 106 months, the occurrence of 357 CVD deaths and 1292 all-cause deaths were found. Baseline characteristics based on TyG index quartiles are shown in **Table 1**. Participants with higher TyG levels were more likely to be male, Mexican American, lower levels of education and PIR, higher proportions of smokers and alcohol drinkers, higher levels of cholesterol and GGT, and higher proportions of diabetes(all P < 0.05). Furthermore, there were also statistically significant differences across the TyG index quartiles in terms of angina, heart failure, stroke, and the use of oral antihypertensive medications and diuretics.

**Table 1:** Baseline characteristics according to triglyceride-glucose index quartile.

TyG quartile	Q1 (5.97-7.91)	Q2 (7.91-8.26)	Q3 (8.26-8.59)	Q4 (8.59-10.62)	P-value
Gender					<0.01
Male	676 (44.10%)	686 (44.78%)	739 (48.21%)	826 (53.88%)	
Female	857 (55.90%)	846 (55.22%)	794 (51.79%)	707 (46.12%)	
Age (Years)	61 (48-71)	60 (46-69)	60 (46-70)	58 (46-68)	<0.01
Race					<0.001
Mexican American	178 (11.61%)	282 (18.41%)	301 (19.63%)	386 (25.18%)	
Other Hispanic	85 (5.54%)	144 (9.40%)	125 (8.15%)	132 (8.61%)	
Non-Hispanic White	673 (43.90%)	756 (49.35%)	827 (53.95%)	742 (48.40%)	
Non-Hispanic Black	507 (33.07%)	257 (16.78%)	178 (11.61%)	167 (10.89%)	
Other Race	90 (5.87%)	93 (6.07%)	102 (6.65%)	106 (6.91%)	
Education					<0.01
Less than high school	386 (25.18%)	458 (29.90%)	462 (30.14%)	532 (34.70%)	
High or equivalent	405 (26.42%)	347 (22.65%)	409 (26.68%)	380 (24.79%)	
College or above	742 (48.40%)	727 (47.45%)	662 (43.18%)	621 (40.51%)	
PIR					0.02
≤1	304 (19.83%)	298 (19.45%)	282 (18.40%)	342 (22.31%)	
>1, ≤3	672 (43.84%)	663 (43.28%)	723 (47.16%)	694 (45.27%)	
>3	557 (36.33%)	571 (37.27%)	528 (34.44%)	497 (32.42%)	
Drinking					<0.01
Never	591 (38.55%)	552 (36.03%)	592 (38.62%)	613 (39.99%)	
Every day or nearly every day	209 (13.63%)	271 (17.69%)	261 (17.03%)	264 (17.22%)	
3 to 4 times a week	172 (11.22%)	209 (13.64%)	196 (12.79%)	206 (13.44%)	
1 to 2 times a week	394 (25.70%)	353 (23.04%)	369 (24.07%)	323 (21.07%)	
Less than once a week	167 (10.89%)	147 (9.60%)	115 (7.50%)	127 (8.28%)	
Smoking status					<0.01
Never	801 (52.25%)	770 (50.26%)	712 (46.44%)	699 (45.60%)	
Now	236 (15.39%)	299 (19.52%)	299 (19.50%)	323 (21.07%)	
Former	496 (32.35%)	463 (30.22%)	522 (34.05%)	511 (33.33%)	
LDL	2.79 (2.20-3.39)	3.05 (2.42-3.67)	3.13 (2.48-3.75)	2.95 (2.30-3.67)	<0.01
TC	4.63 (3.96-5.35)	4.99 (4.32-5.77)	5.25 (4.55-5.97)	5.30 (4.63-6.18)	<0.01
ALT	20 (16-27)	22 (17-30)	23 (17-31)	25 (19-35)	<0.01
AST	22 (19-27)	23 (19-27)	23 (20-28)	24 (20-29)	<0.01
Scr	77.79 (63.65-93.70)	75.14 (61.90-89.28)	76.91 (62.76-89.28)	75.14 (61.88-91.05)	<0.01
eGFR	97.33 (88.97-107.09)	99.32 (91.24-108.67)	98.61 (90.68-108.60)	99.94 (91.05-108.67)	<0.01
GGT	21 (15-31)	23 (17-33)	25 (18-37)	30 (21-47)	<0.01
Uric acid	345.00 (291.50-398.50)	345.00 (297.40-404.50)	356.90 (297.40-410.40)	350.90 (291.50-416.40)	<0.01
Hypertension					<0.01
Yes	1285 (83.82%)	1154 (75.33%)	1035 (67.51%)	1034 (67.45%)	
No	248 (16.18%)	378 (24.67%)	498 (32.49%)	499 (32.55%)	
Diabetes					<0.01
Yes	494 (32.22%)	408 (26.63%)	498 (32.49%)	860 (56.10%)	
No	1038 (67.71%)	1123 (73.30%)	1034 (67.45%)	672 (43.84%)	
CHD					0.08
Yes	102 (6.71%)	90 (5.90%)	120 (7.89%)	121 (7.92%)	
No	1419 (93.29%)	1436 (94.10%)	1400 (92.11%)	1406 (92.08%)	
MI					0.16
Yes	124 (8.12%)	93 (6.09%)	114 (7.45%)	117 (7.64%)	
No	1403 (91.88%)	1434 (93.91%)	1417 (92.55%)	1415 (92.36%)	
Angina					0.03
Yes	82 (5.37%)	54 (3.53%)	84 (5.50%)	82 (5.37%)	
No	1444 (94.63%)	1474 (96.47%)	1443 (94.50%)	1445 (94.63%)	
HF					0.03
Yes	105 (6.89%)	70 (4.58%)	79 (5.19%)	92 (6.02%)	
No	1420 (93.11%)	1459 (95.42%)	1444 (94.81%)	1436 (93.98%)	
Stroke					<0.01
Yes	113 (7.39%)	87 (5.69%)	70 (4.57%)	81 (5.30%)	
No	1416 (92.61%)	1443 (94.31%)	1463 (95.43%)	1448 (94.70%)	
Antihypertensive Medications					<0.01
Never	332 (21.66%)	382 (24.93%)	397 (25.90%)	361 (23.55%)	
Yes	848 (55.32%)	750 (48.96%)	711 (46.38%)	733 (47.81%)	
Unknown or missing	353 (23.03%)	400 (26.11%)	425 (27.72%)	439 (28.64%)	
Diuretics					<0.01
Never	332 (21.66%)	382 (24.93%)	397 (25.90%)	361 (23.55%)	
Yes	424 (27.66%)	343 (22.39%)	349 (22.77%)	332 (21.66%)	
Unknown or missing	777 (50.68%)	807 (52.68%)	787 (51.34%)	840 (54.79%)	
Statins					0.06
Never	332 (21.66%)	382 (24.93%)	397 (25.90%)	361 (23.55%)	
Yes	468 (30.53%)	407 (26.57%)	411 (26.81%)	433 (28.25%)	
Unknown or missing	733 (47.81%)	743 (48.50%)	725 (47.29%)	739 (48.21%)	

Data were presented as median (Interquartile range) or n (%).

TyG, Triglyceride-glucose index; BMI, Body Mass Index; PIR, Poverty Impact Ratio; LDL, Low-density lipoprotein; TC, Total Cholesterol; ALT, Alanine aminotransferase; AST, Aspartate transaminase; Scr, Serum creatinine; eGFR, Estimated glomerular filtration Rate; GGT, gamma-glutamyl transferase; CHD, Coronary heart disease; MI, Myocardial infarction; HF, Heart failure.

### 3.2 Associations between TyG index and mortality in MeS

In the proportional risk assumption, Schoenfeld residuals was used to assess the constancy of the hazard ratios over time. The results of this testing did not indicate any significant violations of the proportional hazards assumption (P>0.05, **Supplementary Figure 1**). **Table 2** shows the association between TyG index and CVD mortality and all-cause mortality in population with MeS. After adjusting for multiple variables, TyG index was statistically positively associated with CVD mortality (HR=1.57, 95%CI: 1.23, 1.99) and all-cause mortality (HR=1.27, 95%CI: 1.11, 1.43) in MeS population. In addition, when the levels of TyG index was divided into quartiles, it was found that the Q4 group significantly increased the CVD mortality (HR=1.20, 95%CI: 1.01, 1.43) and all-cause mortality (HR=1.35, 95%CI: 1.01, 1.91), respectively, compared with the lowest level of the Q1 group. But the P value for trend was not statistically significant in the relationship between TyG index quartiles and CVD mortality (P>0.05).

**Table 2:** Relationship between TyG index and cardiovascular disease mortality and all-cause mortality.

	No. of death	Model 1	Model 2	Model 3
CVD mortality				
TyG index		1.24 (1.01, 1.52) 0.03	1.34 (1.09, 1.65) 0.01	1.57 (1.23, 1.96) <0.01
Q1	75 (4.89%)	1.0	1.0	1.0
Q2	83 (5.42%)	0.84 (0.62, 1.16) 0.29	0.99 (0.72, 1.35) 0.93	1.12 (0.87, 1.53) 0.67
Q3	101 (6.59%)	1.05 (0.78, 1.41) 0.77	1.11 (0.82, 1.49) 0.50	1.23 (0.91, 1.72) 0.28
Q4	98 (6.39%)	1.06 (0.79, 1.44) 0.69	1.21 (0.89, 1.63) 0.22	1.35 (1.01, 1.91) 0.04
P for trend		0.36	0.15	0.07
All-cause mortality				
TyG index		1.14 (1.02, 1.26) 0.01	1.22 (1.09, 1.36) <0.01	1.27 (1.11, 1.43) <0.01
Q1	288 (18.79%)	1.0	1.0	1.0
Q2	306 (19.97%)	0.81 (0.69, 0.96) 0.01	0.93 (0.79, 1.09) 0.38	0.99 (0.84, 1.17) 0.89
Q3	337 (21.98%)	0.91 (0.78, 1.07) 0.24	0.96 (0.82, 1.13) 0.63	0.98 (0.83, 1.16) 0.80
Q4	361 (23.55%)	1.02 (0.88, 1.19) 0.77	1.15 (0.98, 1.34) 0.08	1.20 (1.01, 1.43) 0.03
P for trend		0.34	0.06	0.04

Model 1 adjust for: None.

Model 2 adjust for: Age, gender.

Model 3 adjust for: Age, gender, race, education, poverty impact ratio, drinking, smoking, total cholesterol, low-density lipoprotein, alanine aminotransferase, aspartate transaminase, estimated glomerular filtration rate, heart failure, stroke, coronary heart disease, angina, myocardial infarction, oral antihypertensive medications, diuretics, and statins

TyG, Triglyceride-Glucose; HR, Hazard ratio; CI, Confidence interval

### 3.3 Non-linear relationship between TyG index and mortality in MeS

**Figure 2:**
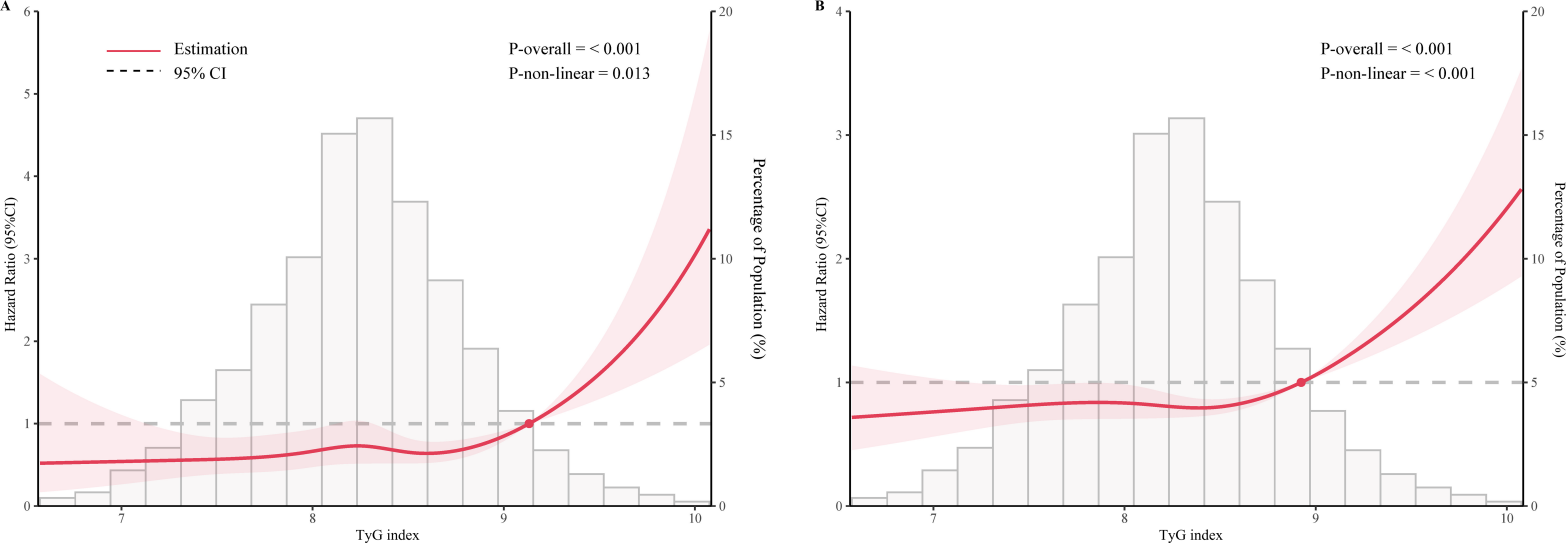
Restricted cubic spline curve for the association of TyG index with mortality after adjusting for all variables. **(A)** Association between TyG index and cardiovascular disease mortality in the metabolic syndrome population. **(B)** Association between TyG index and all-cause mortality in the metabolic syndrome population.

As shown in **Figure 2**, after adjustment for multiple potential confounders, the nonlinear associations between TyG index and CVD and all-cause mortality were statistically significant (P<0.05). We used RCS and segmented Cox proportional hazards regression models to assess the relationship. **Figures 2A, B** show a J-type relationship between TyG index and CVD mortality and all-cause mortality, with optimal inflection point values of 9.13 and 8.92, respectively (**Table 3**). When TyG index higher than the inflection point, the risk of CVD and all-cause mortality progressively increased, and the HR (95%CI) was 4.21 (95%CI: 1.82, 9.78) and 2.93 (95%CI:2.05, 4.18) (**Table 3**).

**Table 3:** Threshold effect analysis of TyG index on all-cause and CVD mortality.

	Adjusted HR (95% CI)	P-value
CVD mortality		
Inflection point	9.13	
TyG index < 9.13	1.27 (0.97, 1.66)	0.08
TyG index ≥ 9.13	4.21 (1.82, 9.78)	<0.01
P for Log- likelihood ratio	0.02	
All-cause mortality		
Inflection point	8.92	
TyG index < 8.92	1.05 (0.91, 1.22)	0.48
TyG index ≥ 8.92	2.93 (2.05, 4.18)	< 0.01
P for Log- likelihood ratio	< 0.01	

Cox proportional hazards models were used to estimate HR and 95% CI. Adjusted for age, gender, race, education, poverty impact ratio, drinking, smoking, total cholesterol, low-density lipoprotein, alanine aminotransferase, aspartate transaminase, estimated glomerular filtration rate, heart failure, stroke, coronary heart disease, angina, myocardial infarction, oral antihypertensive medications, diuretics, and statins.

### 3.4 Analysis of the relationship between TyG index and CVD mortality based on competing risks model

**Figure 3:**
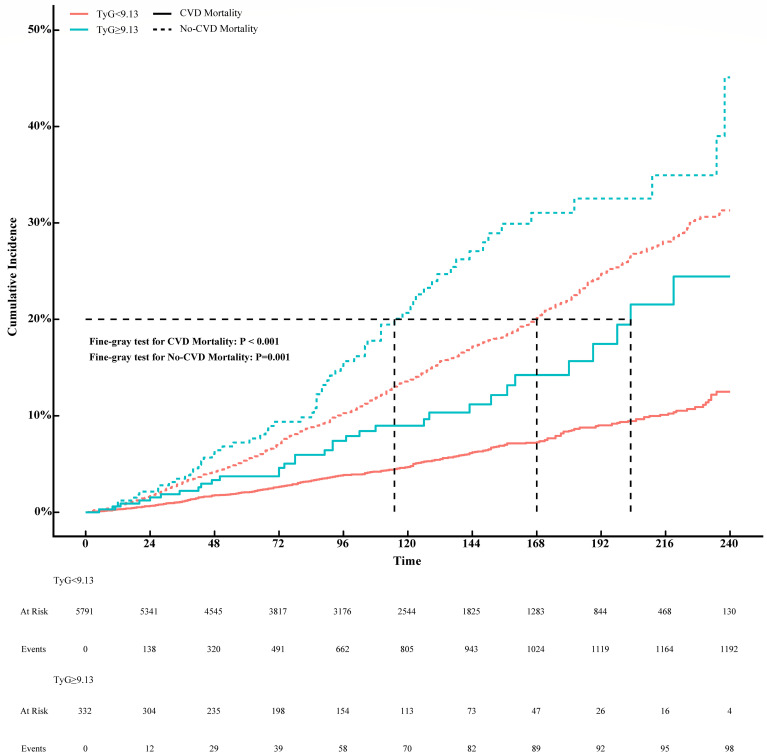
Association between TyG index and CVD mortality with non-CVD mortality as a competing risk (Fine-Gray competing risk model).

To further investigate the prognostic impact of the TyG index on CVD mortality in the MeS population, we divided the TyG index into two groups of <9.13 and ≥9.13 according to the inflection point. Non-CVD mortality was considered as competing event and competing risk analyses were performed by the Fine-Gray competing risk model. The cumulative incidences of 1, 3, 5 and 10 years in TyG index <9.13 were 0.31%, 1.10%, 2.10% and 4.70%, while 0.60%, 2.20%, 3.70% and 9.00% in TyG index ≥9.13 in CVD motality (P < 0.001, **Supplementary Table 2**). As shown in **Figure 3**, the cumulative CVD mortality incidences were higher in MeS with TyG index >9.13 than that with TyG index <9.13 (Fine-Gray P = 0.001). However, the corresponding incidences of competing risk events were also higher in MeS with TyG index ≥9.13 than that in TyG index <9.13 in no-CVD mortality (1.20%, 3.50%, 7.20%, 21.00% versus 0.67%, 3.10%, 5.60%, 14.00%; Fine-Gray P = 0.001, **Supplementary Table 2**).

### 3.5 Mediating effect of oxidative stress

**Figure 4:**
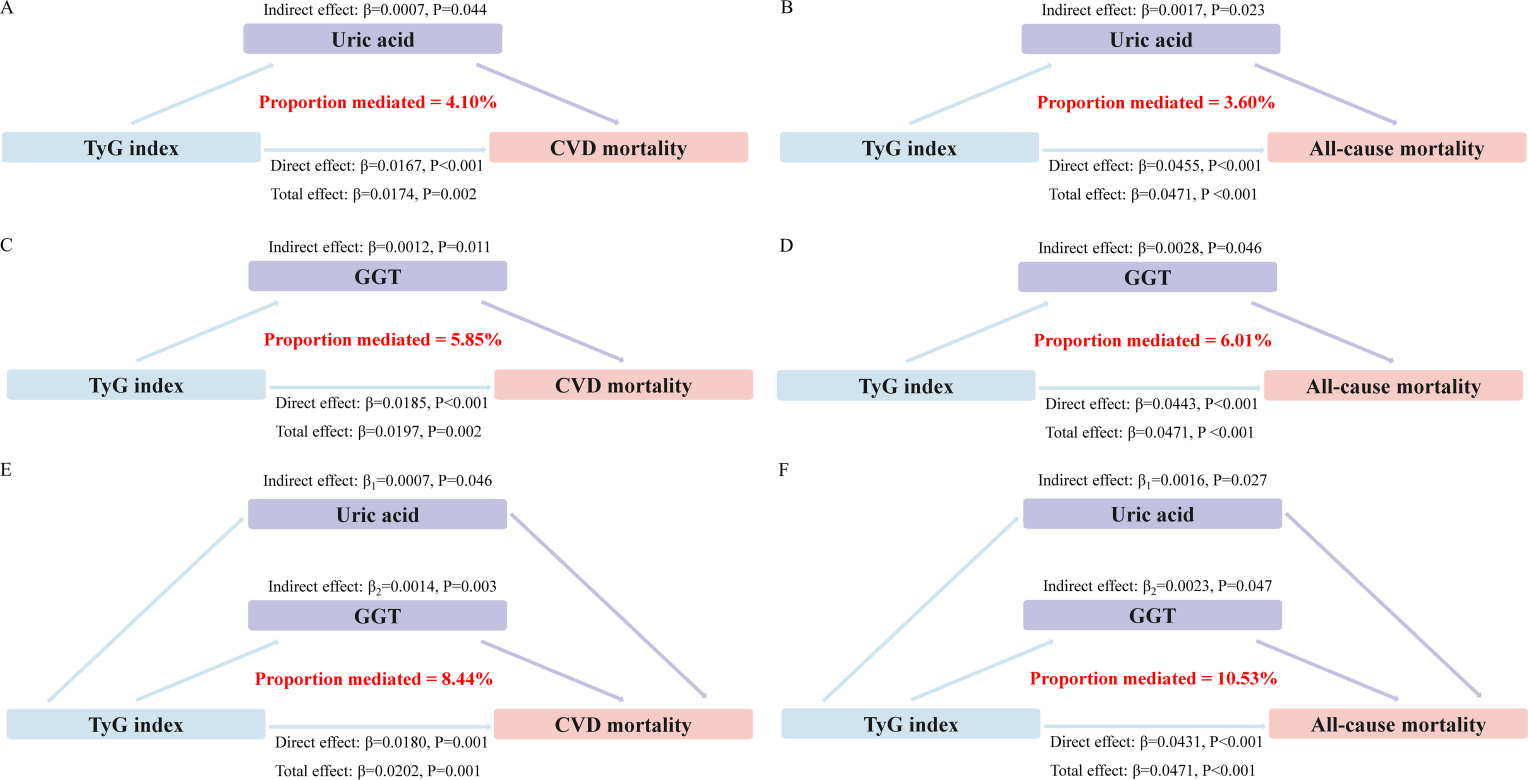
Estimated proportion of the association between TyG index and mortality mediated by oxidative stress factor. **(A)** The mediating effect of uric acid in CVD mortality. **(B)** The mediating effect of uric acid in all-cause mortality. **(C)** The mediating effect of GGT in CVD mortality. **(D)** The mediating effect of GGT in all-cause mortality. **(E)** Joint mediating effects of uric acid and GGT in CVD mortality. **(F)** Joint mediating effects of uric acid and GGT in all-cause mortality.

Mediation analyses showed that that the levels of GGT and uric acid had significant mediating effects on the association between TyG index and mortality (**Figure 4**). Notably, GGT and uric acid mediated 5.85% and 4.10% of the association between TyG index and CVD mortality, with a combined mediation proportion of 10.53% (P<0.05). Similarly, mediating effects of GGT and uric acid on the relationship between TyG index and all-cause mortality were also observed to be 6.01% and 3.60%, respectively, with a combined mediating effect of 8.44%(P<0.05).

Subsequently, we conducted a bootstrap test to evaluate the significance of the mediation effects. The results indicated that the 95%CIs from the bootstrap analysis did not include zero, suggesting that the mediation effects were statistically significant (**Supplementary Table 3**). This finding supports the role of oxidative stress markers as significant mediators in the relationship between the TyG index and mortality.

### 3.6 Sensitivity analysis

To further demonstrate the robustness of our results, we conducted the sensitivity analysis in the MeS population with CVD (Sensitivity-1), with after excluding participants who died within the first 2 years of follow-up (Sensitivity-2), with who were taking oral antihypertensive medications, diuretics, or statins (Sensitivity-3). The results were found that TyG index still showed a J-shaped relationship with CVD death and all-cause mortality, demonstrating the strong robustness of our results (**Table 4**).

**Table 4:** Sensitivity analysis of the effect of TyG index on CVD and all-cause mortality.

	Sensitivity-1 (HR, 95%CI, P-value)	Sensitivity-2 (HR, 95%CI, P-value)	Sensitivity-3 (HR, 95%CI, P-value)
CVD mortality			
Inflection point=9.13			
TyG index < 9.13	1.08 (0.70, 1.69) 0.72	1.26 (0.94, 1.69) 0.12	1.32 (0.98, 1.79) 0.07
TyG index ≥ 9.13	8.52 (2.24, 32.37) <0.01	3.63 (1.58, 8.34) <0.01	4.75 (1.84, 12.26) <0.01
P for Log-likelihood ratio	0.02	0.04	0.03
All-cause mortality			
Inflection point=8.92			
TyG index < 8.92	0.84 (0.65, 1.09) 0.19	1.06 (0.91, 1.24) 0.43	0.99 (0.83, 1.18) 0.90
TyG index ≥ 8.92	3.35 (1.58, 7.13) <0.01	2.95 (2.03, 4.30) <0.01	2.99 (1.94, 4.61) <0.01
P for Log-likelihood ratio	<0.01	<0.01	<0.01

Sensitivity-1: including 894 metabolic syndrome population with cardiovascular disease. Adjusted for age, race, education, poverty impact ratio, body mass index, drinking, smoking, total cholesterol, low-density lipoprotein, alanine aminotransferase, aspartate transaminase, estimated glomerular filtration rate, oral antihypertensive medications, diuretics, and statins.

Sensitivity-2: After excluding participants who died within the first two years of follow-up, 5645 participants were included. Adjusted for age, race, education, poverty impact ratio, body mass index, drinking, smoking, total cholesterol, low-density lipoprotein, alanine aminotransferase, aspartate transaminase, Age, gender, race, education, poverty impact ratio, drinking, smoking, total cholesterol, low-density lipoprotein, alanine aminotransferase, aspartate transaminase, estimated glomerular filtration rate, heart failure, stroke, coronary heart disease, angina, myocardial infarction, oral antihypertensive medications, diuretics, and statins.

Sensitivity-3: including 3,413 individuals with metabolic syndrome who were taking oral antihypertensive medications, diuretics, or statins. Adjusted for age, race, education, poverty impact ratio, body mass index, drinking, smoking, total cholesterol, low-density lipoprotein, alanine aminotransferase, aspartate transaminase, estimated glomerular filtration rate, heart failure, stroke, coronary heart disease, angina and myocardial infarction.

Furthermore, based on Model 3, we calculated the E-Values for the relationship between the TyG index and CVD mortality and all-cause mortality, which were 2.52 and 1.86, respectively. This indicates that any confounding factor associated with the TyG index would need to have a HR of at least 2.52 for CVD mortality and 1.86 for all-cause mortality to reduce the HR to below 1. Given that many known confounding factors have been adjusted for, it is unlikely that a single unmeasured residual confounder would have an association of this magnitude.

## 4 Discussion

This is a large prospective cohort study based on NHANES 1999-2018, which found that oxidative stress mediated the association of TyG index with CVD and all-cause mortality in the MeS population. Our findings suggested a J-shaped association between TyG index and CVD and all-cause mortality in MeS, with the inflection point of 9.13 for CVD mortality and 8.92 for all-cause mortality. In addition, oxidative stress markers, GGT and uric acid, play a mediating role in the association between TyG index and CVD mortality and all-cause mortality, with a combined mediation proportion of 10.53% and 8.44%, respectively. This is the first study to assess the oxidative stress mediated relationship between TyG index and mortality in MeS population.

Currently, the diagnostic criteria for MeS are derived from several sources, including the NCEP ATP III criteria (16), the International Diabetes Federation (IDF) criteria (22), the World Health Organization (WHO) criteria (23), and the European Group for the Study of Insulin Resistance (EGIR) criteria (24). The NCEP ATP III criteria were selected due to their prevalence and historical use within the United States (25). These criteria have been extensively utilized in numerous epidemiological and clinical studies conducted within the U.S., providing a robust framework for comparison and continuity in research. Moreover, the use of the NCEP ATP III criteria was influenced by the specific demographic and clinical context of our study population. The U.S.-based nature of our cohort and the well-established use of the NCEP ATP III criteria in this population provided a compelling rationale for the choice.

The TyG index has emerged as a significant predictor of cardiovascular and endocrine diseases (26, 27). The TyG index has been found associated with an increased risk of developing type 2 diabetes mellitus (T2DM) (6), as well as adverse cardiovascular outcomes (28). In hypertensive populations, insulin resistance (assessed using the TyG index) was significantly associated with cognitive and physical impairments (29). In addition, it has been shown that elevated levels of the TyG index predict the progression of coronary artery calcification and the incidence of cardiovascular events independently of traditional risk factors such as hypertension, hypercholesterolemia and smoking (30). The TyG index reflects the combined effects of hyperglycemia and dyslipidemia, therefore, it may be an important tool for prognostic prediction in patients with MeS. Currently, there are few studies on the relationship between TyG index and prognosis in populations with MeS, and the mechanisms involved are unclear.

Our study found TyG index showed a J-shaped relationship with CVD and all-cause mortality in the MeS population. In fact, the TyG index has been shown a non-linear relationship with cardiovascular death and all-cause mortality in several populations. Zhang et al (31) found a U-shaped association was observed between the baseline TyG index with CVD and all-cause mortality in CVD patients with diabetes or pre-diabetes. Interestingly, this U-shaped association appears to be confirmed in the general population (32). Wen et al (33) demonstrated a strong U/J-shaped relations were observed for all-cause and CVD death in familial hypercholesterolemia. These discrepancies may be due to differences in study populations, sample sizes and methodologies between studies. And the dynamics of the relationship between TyG and mortality may be affected by underlying metabolic conditions in different populations. In another study, the TyG index was reported to be positively associated with all-cause mortality in critically ill patients (34). It emphasizes the need to consider the clinical context and patient populations when interpreting the TyG index’s role in prognostic prediction. It is also possible that the mechanisms linking the TyG index to outcomes may vary across different ethnicities and geographical locations, as suggested by a recent review highlighting the need for a more global perspective in evaluating the application value of TyG index (35). However, the mechanism of this association remains unknown.

This mechanism regarding TyG index and prognosis in the MeS population is complex and involves multiple interrelated pathways, including oxidative stress, inflammation, endothelial dysfunction and metabolic dysregulation (13–15). Of these, oxidative stress is thought to play an important role. The TyG index is a composite indicator of insulin resistance, which is one of the features of the metabolic syndrome. The interplay between insulin resistance and oxidative stress is complex and bidirectional. Firstly, insulin resistance can lead to increased production of reactive oxygen species (ROS) (36), which in turn exacerbates insulin resistance through various mechanisms, including impaired insulin signaling (37) and inflammation (38). The activation of inflammatory pathways, as evidenced by elevated levels of TNF-α and other cytokines, further contributes to the vicious cycle of insulin resistance and oxidative stress (2, 39). Secondly, oxidative stress also plays a critical role in endothelial dysfunction (40), a key early event in the development of atherosclerosis. By impairing nitric oxide (NO) bioavailability and promoting pro-inflammatory responses, oxidative stress contributes to the progression of CVD (41). Additionally, the pro-apoptotic effects of oxidative stress can lead to tissue damage and functional impairment, increasing the risk of CVD events and mortality (42). Thus, mediation analyses were performed to elucidate the critical role of oxidative stress in the relationship between TyG index and mortality. By analyzing the mediating role of uric acid and GGT, it was shown that these oxidative stress markers may be active mediators in the pathophysiological cascade leading to adverse outcomes.

GGT is an enzyme primarily found in the liver and is often considered a marker of hepatic oxidative stress and inflammation (43). Its activity increases in response to cellular damage caused by reactive oxygen species (ROS) and other pro-oxidative agents (44). Elevated GGT levels reflect a state of increased glutathione (GSH) consumption, which is a critical antioxidant defense mechanism in the body (45). GSH is depleted as it neutralizes ROS, leading to an upregulation of GGT activity to maintain cellular redox balance. In the MeS, GGT may indicate the presence of a pro-oxidative environment that accompanies IR and its associated metabolic derangements (46). This environment can exacerbate endothelial dysfunction, promote atherosclerosis, and ultimately increase the risk of CVD and all-cause mortality.

Uric acid, a product of purine metabolism, serves as another biomarker of oxidative stress (47). Uric acid acts as an endogenous antioxidant by scavenging ROS and protecting against oxidative damage. However, excessive oxidative stress can tip the balance, leading to elevated uric acid levels as the body’s antioxidant defenses become overwhelmed. High levels of uric acid have been linked to hypertension, metabolic syndrome, and gout, all of which are risk factors for CVD (48). In the context of the TyG index, uric acid may indicate the cumulative burden of oxidative stress, which can result in vascular damage, increased cardiovascular events, and higher mortality rates.

Our study has important clinical implications. Strategies such as maintaining a balanced diet rich in antioxidants, engaging in regular physical activity, managing weight, quitting smoking, moderating alcohol consumption, reducing stress, and getting adequate sleep can all help to lower oxidative stress levels. These interventions target not only the TyG index but also the broader metabolic derangements associated with metabolic syndrome. This provides the basis for reducing the burden of cardiovascular disease and improving overall health. The potential for lifestyle modifications to reduce oxidative stress and improve clinical outcomes is a promising area for future research and intervention. However, our study has some limitations. Firstly, there was too much missing data on exposure variables, outcome variables and covariates. This missing data could introduce bias, potentially distorting the results away from an accurate representation of the true population, and may have reduced the statistical power to detect significant effects or differences. Secondly, as is common in many studies, the TyG index was calculated based on a single measurement of TG and FPG, which does not reflect dynamic changes during long-term follow-up and may impact the prognosis of MeS. It is important to recognize that individuals who have been identified with abnormalities in blood glucose or lipids may have taken medical interventions to correct these conditions. Consequently, the predictive accuracy of the TyG index for long-term outcomes may be compromised in such cases. The possibility of misclassification due to this single measurement could potentially affect the results of our study, as it may not fully represent the chronic exposure to the risk factors associated with MeS. Future studies should incorporate multiple measurements of the TyG index over time to more accurately reflect chronic metabolic changes and their prognostic implications. Fourth, although we have attempted to control for confounding factors, it is inevitable that some residual confounding remains due to the inability to account for all disease-related measurements that may influence outcomes. As a supplementary explanatory approach, we further calculated the E-values based on the results of Model 3. This analysis indicates that our findings have relative stability, as most unknown confounders are unlikely to reach the high estimated HRs of 2.52 for CVD mortality and 1.86 for all-cause mortality. Finally, our study, conducted in a US population, may not fully capture the ethnic variability that exists in the global context. It is recognized that ethnic differences may influence the association of the TyG index with oxidative stress and mortality outcomes, as suggested by recent research highlighting genetic and lifestyle differences between ethnic groups. The generalizability of our findings may therefore be limited to populations with similar ethnic backgrounds. Future studies should consider the inclusion of diverse ethnic groups to better understand these associations and increase the global applicability of the findings.

This study provides evidence that the TyG index is associated with mortality in patients with MeS, showing a J-shaped relationship. It suggests the importance of managing the TyG index within an optimal range to potentially reduce the risk of cardiovascular disease and all-cause mortality. The study also points to oxidative stress as an important mediator, suggesting potential targets for therapeutic intervention. However, our study may have been confounded by potential confounders, and the generalizability of the findings may be limited by the specific populations studied. Future research should aim to confirm these associations in different populations and over time, and explore the mechanisms by which oxidative stress influences the prognostic value of the TyG index.

## Data Availability

The original contributions presented in the study are included in the article/**Supplementary Material**. Further inquiries can be directed to the corresponding author.
